# GenoType MTBDR*plus* Assay for Rapid Detection of Multidrug Resistance in *Mycobacterium tuberculosis*: A Meta-Analysis

**DOI:** 10.1371/journal.pone.0150321

**Published:** 2016-03-02

**Authors:** Yuanyuan Bai, Yueling Wang, Chunhong Shao, Yingying Hao, Yan Jin

**Affiliations:** Department of Clinical Laboratory, Shandong Provincial Hospital Affiliated to Shandong University, Jinan, PR China; Indian Institute of Science, INDIA

## Abstract

**Background:**

There is an urgent demand for rapid and accurate drug-susceptibility testing for the detection of multidrug-resistant tuberculosis. The GenoType MTBDR*plus* assay is a promising molecular kit designed for rapid identification of resistance to first-line anti-tuberculosis drugs, isoniazid and rifampicin. The aim of this meta-analysis was to evaluate the diagnostic accuracy of GenoType MTBDR*plus* in detecting drug resistance to isoniazid and rifampicin in comparison with the conventional drug susceptibility tests.

**Methods:**

We searched PubMed, EMBASE, and Cochrane Library databases to identify studies according to predetermined criteria. A total of 40 studies were included in the meta-analysis. QUADAS-2 was used to assess the quality of included studies with RevMan 5.2. STATA 13.0 software was used to analyze the tests for sensitivity, specificity, positive likelihood ratio, negative likelihood ratio, diagnostic odds ratio, and area under the summary receiver operating characteristic curves. Heterogeneity in accuracy measures was tested with Spearman correlation coefficient and Chi-square.

**Results:**

Patient selection bias was observed in most studies. The pooled sensitivity (95% confidence intervals were 0.91 (0.88–0.94) for isoniazid, 0.96 (0.95–0.97) for rifampicin, and 0.91(0.86–0.94) for multidrug-resistance. The pooled specificity (95% CI) was 0.99 (0.98–0.99) for isoniazid, 0.98 (0.97–0.99) for rifampicin and 0.99 (0.99–1.00) for multidrug-resistance, respectively. The area under the summary receiver operating characteristic curves ranged from 0.99 to 1.00.

**Conclusion:**

This meta-analysis determined that GenoType MTBDR*plus* had good accuracy for rapid detection of drug resistance to isoniazid and/or rifampicin of *M*. *tuberculosis*. MTBDR*plus* method might be a good alternative to conventional drug susceptibility tests in clinical practice.

## Introduction

Tuberculosis (TB) is one of the most serious infectious diseases and a main cause of morbidity and mortality in developing countries [1]. The World Health Organization (WHO) estimated that approximately 450,000 people developed multidrug-resistant TB (MDR-TB), and 170,000 MDR-TB-related deaths occurred in 2012 worldwide [2]. MDR-TB which is defined as resistance *in vitro* to first-line drugs, rifampicin (RIF) and isoniazid (INH), has posed a great challenge to the successful control of TB in the world [3, 4]. Treatment of MDR-TB is costly, complicated, with less effective therapies and is associated with treatment failures, relapses, and poor clinical outcomes [5, 6].

Conventional phenotypic drug susceptibility testing (DST) has been recommended as the gold standard by WHO, including tests that are performed on solid media (proportion method (PM) on Lowenstein-Jensen (L-J) or Middlebrook 7H10/7H11 agar) and liquid systems (BACTEC 460 and BACTEC MGIT 960) [7]. However, conventional methods have some limitations. Solid media-based DST have a long turnaround time, which can take longer than 2 months, which may result in delayed proper treatment, increasing risk of treatment failure, and continuing transmission of drug-resistance [8]. Liquid systems-based DST are sensitive and faster than solid media-based DST (they take up to 25–45 days), but are more costly; due to the increased technical complexity, there is a lack of appropriately-trained technicians [9]. Therefore, there is an urgent need for the development of rapid and accurate DST for MDR-TB which is able to avoid clinical deterioration, improve treatment regimen, and interrupt further transmissions.

The technological advancement of molecular biotechnologies has been of interest for DSTs that target MDR-TB. The WHO endorsed the use of molecular line-probe assays (LiPAs) for MDR-TB screening in 2008 [10]. The GenoType MTBDR*plus* assay (Hain Lifescience, Nehren, Germany) is a commercially available LiPA that combines detection of *M*. *tuberculosis* complex with prediction of resistance to RIF and INH, including mutations in the 81-bp hotspot region of *rpoB*, at codon 315 of the *katG* gene and in the *inhA* promoter region [11]. This assay is comprised of DNA extraction, multiplex polymerase chain reaction (PCR), reverse hybridization, and resistance gene mutations detection, all of which can be completed within 8 hours. Two previously published meta-analyses found that GenoType MTBDR*plus* assay had good diagnostic accuracy compared to conventional DST [12, 13]; however, those analyses were limited by the small number of included studies and significant unexplained heterogeneity in accuracy measures. One of those studies only evaluated the assay on clinical specimens, therefore could not fully assess the clinical application of MTBDR*plus* assay [13].

Several previous studies have examined the performance of the GenoType MTBDR*plus* assay when testing for RIF and INH resistance based on the related genes; however, the sensitivity and specificity results have been inconsistent. In the present study, a new meta-analysis was performed to comprehensively evaluate the overall diagnostic accuracy of the GenoType MTBDR*plus* assay in detecting drug resistance of RIF and INH compared with conventional DST.

## Methods

We followed the Preferred Reporting Items for Systematic Reviews and Meta-Analyses (PRISMA) guidelines in our study. We registered the review in PROSPERO (crd.york.ac.uk CRD42015027271).

### Literature Search

Original articles published in English up to the end of July 2015 were searched in PubMed, EMBASE, and Cochrane Library databases by two investigators (Y. Bai and Y. Jin). The search terms used were as follows: (Tuberculosis OR *Mycobacterium tuberculosis*) AND (Hain Life Science OR line probe assay OR GenoType MTBDR OR molecular diagnostic techniques). Conference abstracts were included when sufficient data were reported. Reference lists from included studies were also searched.

### Study Criteria

We included studies that evaluated GenoType MTBDR*plus* for detection of drug resistance of *M*. *tuberculosis* to rifampicin (RIF) and/or isoniazid (INH). Included studies should have compared the GenoType MTBDR*plus* with one or more reference standard methods that were recommended by the WHO (including L-J PM, Middlebrook 7H10/7H11 agar, BACTEC 460, and BACTEC MGIT 960). The study report must have had extractable data to fill the 4 cells of a 2 × 2 table for diagnostic tests (true resistant-TR, false resistant-FR, false susceptible-FS, and true susceptible-TS).

Relevant publications were excluded if they were duplicated articles, letters without original data, case reports, editorials, and reviews. Studies with fewer than 10 samples were also excluded to reduce selection bias.

### Data Extraction

The final set of articles was independently assessed by two investigators (Y. Bai and Y. Jin). The full-text of each study was carefully read according to the inclusion criteria to assess whether it should be included. Disagreements were resolved by consensus. Information was extracted on the first author, publication year, country where the study was conducted, specimen type, sample size, gold standard DST used, the number of TR, the number of FR, the number of FS, and the number of TS to each drug. Sensitivity was defined as the proportion of isolates correctly determined as resistant by use of the GenoType MTBDR*plus* compared with gold standard. Specificity was defined as the proportion of isolates correctly determined susceptible by use of the GenoType MTBDR*plus* compared with gold standard.

### Quality of Study Reports

We applied the Quality Assessment of Diagnostic Accuracy Studies (QUADAS-2) to assess the quality of included studies (http://www.bris.ac.uk/quadas/), an updated version of the original software. QUADAS-2 is used in systematic reviews to evaluate the risk of bias and applicability of diagnostic accuracy studies, and consists of four key domains: patient selection, index test, reference standard, and flow and timing. Each domain is assessed for risk of bias and the first three are also evaluated for applicability. Signaling questions were included to assist in judgments about the risk of bias [14]. If the answers to all signaling questions for a domain were “yes,” the risk of bias is judged as “low;” if any signaling question in a domain was “no,” risk of bias is judged as “high.” The unclear bias should only be used if insufficient information was supplied [14]. Applicability was judged as low, high, or unclear with the similar criteria.

### Statistical Analysis

#### Accuracy Estimates

Meta-analyses were performed using two software programs: STATA 13.0 (Stata Corporation, Texas, USA) and Cochrane RevMan 5.2. Sensitivity, specificity, positive likelihood ratio (PLR), negative likelihood ratio (NLR), and diagnostic odds ratio (DOR), forest plots and summary receiver operating characteristic (SROC) curves were analyzed with the STATA 13.0 software, based on the random model effect. Quality of studies was assessed with RevMan 5.2. The SROC curve was used to evaluate the effect of the assay. The area under the curve (AUC) displayed the overall diagnostic accuracy and range between 0 and 1, with higher values indicating better test performance [15].

#### Heterogeneity

Heterogeneity refers to a high degree of variability in accuracy estimates across studies and is often concerned in meta-analyses. We used chi-square test and I^2^ (*P* < 0.05 and I^2^ > 50% indicated significant heterogeneity) to identify heterogeneity [16]. The Spearman correlation coefficient between the logit of sensitivity and logit of 1-specificity was used to assess the threshold/cut off effect, which is a possible cause of variations in sensitivity and specificity among the included studies [15]. Heterogeneity due to factors other than threshold/cut-off effect was tested by visual inspection of the forest plots. The further reasons for heterogeneity of the data were addressed by performing subgroup analyses with the GenoType MTBDR*plus* performed directly on clinical specimens or indirectly on clinical isolates, in either solid or liquid medium.

## Results

### Characteristics of Selected Studies

**Fig 1:**
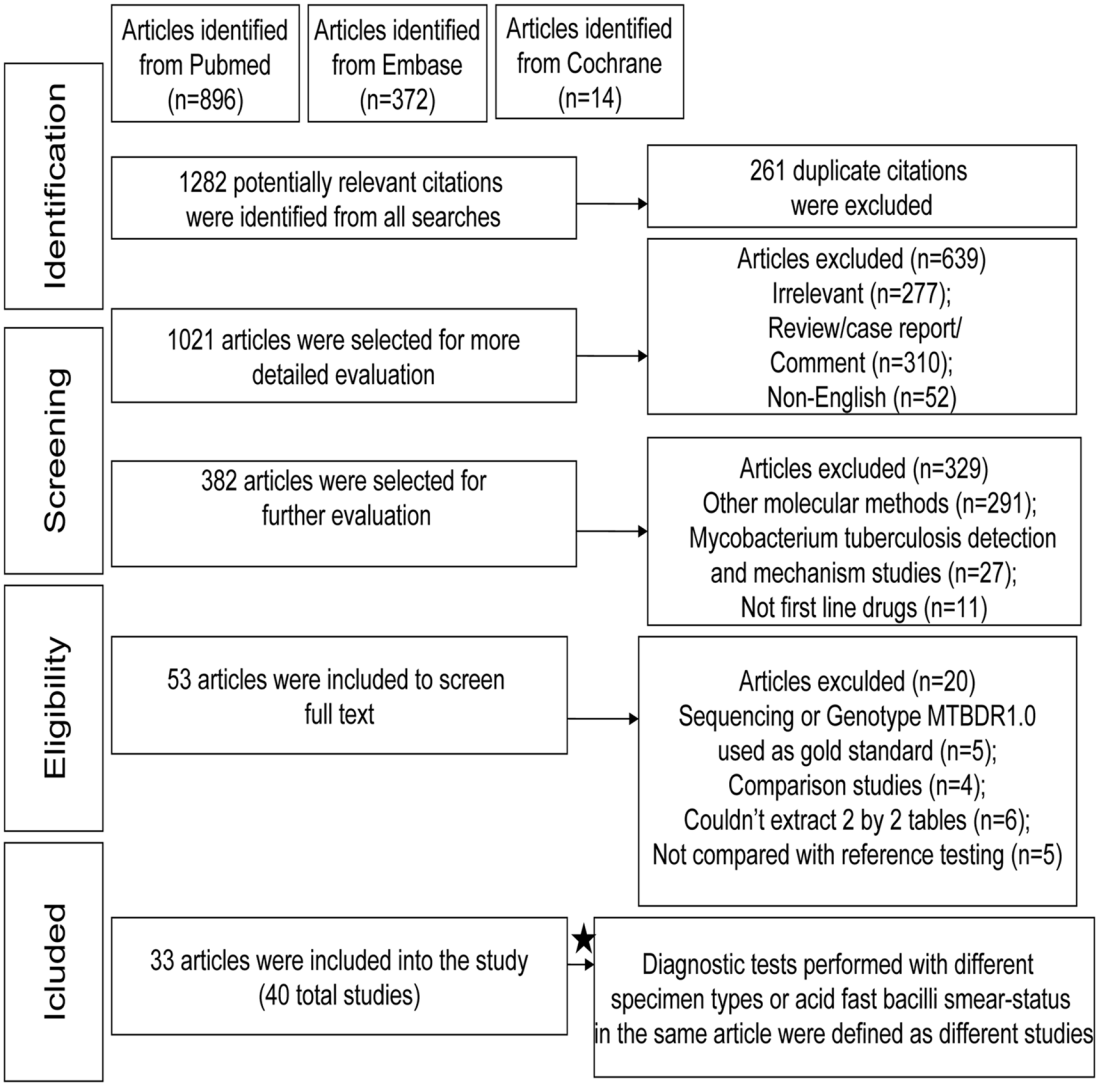
Flow chart of study selection. Of 1282 citations identified, 1229 were excluded after reviewing titles and abstracts. Full-text review of the remaining 53 articles yielded 33 papers meeting eligibility criteria. Because several studies made more than one comparison, there were 40 unique studies.

A flow chart of the study selection process is shown in Fig 1. A total of 1282 potentially relevant citations were identified from all searches. Finally, according to the inclusion and exclusion criteria, 33 eligible articles fulfilled the inclusion criteria and were included in the meta-analysis. The 20 full-text excluded articles were listed in S1 Table with the reasons for exclusion. Because diagnostic tests were performed in different sample types or acid fast bacillus (AFB) smear status occurred in the same article, 40 independent studies (including 7913 samples) were defined in the meta-analysis. Table 1 shows the characteristics of these included studies [17–49]. Among the 40 studies, 23 studies tested clinical specimens (most were AFB smear positive), 14 tested clinical isolates, and the other 3 studies used both. DST was performed based on solid media (L-J PM, agar PM) and liquid systems (BACTEC MGIT 960, BACTEC 460TB). The reference method used was solid medium in 17 studies, liquid medium in another 17 studies, and both solid and liquid medium in 6 studies. Most of the studies were cross-sectional in design.

**Table 1:** Summary of the included studies.

								INH				RIF				MDR			
First author	year	Country	Sample size	Gold standards	Smear status	Specimen type	Study design	TR	FR	FS	TS	TR	FR	FS	TS	TR	FR	FS	TS
Hillemann (17)	2007	Germany	71	MGIT 960 and L-J PM	+	clinical specimens	both	37	0	4	30	30	1	1	39	29	0	2	40
Barnard (18)	2008	South Africa	454	MGIT 960	+	clinical specimens	cross sectional	114	1	7	330	94	2	1	357	85	0	3	372
Causse (19)	2008	Spain	18	MGIT 960	+	clinical specimens	case control	8	0	0	10	9	0	0	9	ND	ND	ND	ND
Causse (19)	2008	Spain	41	MGIT 960		clinical isolates	case control	27	1	0	11	27	0	0	14	ND	ND	ND	ND
Lacoma (20)	2008	Spain	62	Bactec 460		clinical isolates	cross sectional	35	0	13	14	11	0	1	50	ND	ND	ND	ND
Lacoma (20)	2008	Spain	51	Bactec 460	+&−	clinical specimens	cross sectional	28	0	2	21	29	1	0	21	ND	ND	ND	ND
Huang (21)	2009	Taiwan	272	MGIT 960 and Middlebrook 7H10		clinical isolates	case control	198	0	44	30	231	0	11	30	190	0	52	30
Macedo (22)	2009	Portugal	67	BACTEC 460 TB and BacT/ALERT MP Process	+	clinical specimens	cross sectional	23	0	0	43	24	0	0	43	ND	ND	ND	ND
Nikolayevskyy (23)	2009	Germany	149	MGIT 960 and L-J PM	+	clinical specimens	cross sectional	114	6	3	26	103	4	4	38	ND	ND	ND	ND
Albert (24)	2010	Uganda	92	MGIT 960	+	clinical specimens	cross sectional	21	0	5	66	15	4	0	73	12	3	1	76
Anek-vorapong (25)	2010	Thailand	164	MGIT 960	+	clinical specimens	cross sectional	27	0	2	135	19	0	0	145	12	0	1	151
Anek-vorapong (25)	2010	Thailand	50	MGIT 960		clinical isolates	cross sectional	14	0	0	36	6	0	0	44	5	0	0	45
Huyen (26)	2010	Vietnam	110	L-J PM		clinical isolates	case control	50	0	4	52	54	0	4	52	48	0	6	52
Cauwelaert (27)	2011	Madagascar	254	L-J PM	+	clinical specimens	case control	55	4	14	181	47	4	1	202	33	4	7	210
Khadka (28)	2011	Nepal	207	L-J PM		clinical isolates	cross sectional	105	0	2	100	77	0	13	117	70	0	16	121
Rigouts (29)	2011	Tanzanian	269	L-J PM	+	clinical specimens	cross sectional	28	3	23	215	3	4	2	260	3	2	2	262
Imperiale (30)	2012	Argentina	94	MGIT 960	+	both	cross sectional	53	0	9	32	41	0	1	52	ND	ND	ND	ND
Crudu (31)	2012	Moldova	77	Middlebrook 7H11	+	clinical specimens	cross sectional	57	1	1	18	51	1	1	24	ND	ND	ND	ND
Crudu (31)	2012	Moldova	79	Middlebrook 7H11	−	clinical specimens	cross sectional	58	3	4	14	49	1	5	24	ND	ND	ND	ND
Dorman (32)	2012	South Africa	221	MGIT SIRE	+	clinical specimens	cross sectional	18	2	11	190	12	2	2	200	11	1	2	202
Farooqi (33)	2012	Pakistan	108	L-J PM	+	clinical specimens	cross sectional	45	0	14	49	51	1	4	54	ND	ND	ND	ND
Jin (34)	2012	China	237	L-J PM		clinical isolates	case control	126	0	42	69	157	0	11	69	115	0	34	88
Mironova (35)	2012	Baltic States	685	MGIT 960	+	clinical specimens	cross sectional	399	35	19	236	323	35	16	311	ND	ND	ND	ND
Mironova (35)	2012	Baltic States	243	MGIT 960		clinical isolates	cross sectional	85	0	5	153	63	7	1	172	ND	ND	ND	ND
Mironova (35)	2012	Baltic States	304	L-J PM	+	clinical specimens	cross sectional	177	26	9	92	157	23	7	117	ND	ND	ND	ND
Mironova (35)	2012	Baltic States	74	L-J PM		clinical isolates	cross sectional	36	2	1	35	27	10	1	36	ND	ND	ND	ND
Raveendran (36)	2012	India	101	BACT/Alert 3D	+	both	cross sectional	34	1	3	63	27	2	0	72	ND	ND	ND	ND
Tessema (37)	2012	Ethiopia	260	BACT/Alert 3D		clinical isolates	cross sectional	33	2	3	222	15	0	0	245	13	0	0	247
Tukvadze (38)	2012	Georgia	458	MGIT 960 and L-J PM	+	clinical specimens	cross sectional	159	2	16	279	112	4	4	338	109	5	5	339
Ferro (39)	2013	Colombia	222	Middlebrook 7H10		clinical isolates	case control	125	0	7	90	119	2	5	95	114	2	9	96
Lyu (40)	2013	South Korea	428	MGIT 960		both	cross sectional	76	6	5	341	57	4	2	365	51	3	5	369
Maschmann (41)	2013	Brazil	62	L-J PM	+	clinical specimens	cross sectional	29	0	19	14	23	2	5	32	16	0	11	35
Yadav (42)	2013	India	242	L-J PM	+	clinical specimens	cross sectional	86	3	7	146	70	2	1	169	66	0	2	174
Aurin (43)	2014	Bangladesh	277	L-J PM	+	clinical specimens	cross sectional	190	1	1	85	188	1	0	88	186	1	1	89
Chen (44)	2014	China	326	L-J PM	+	clinical specimens	cross sectional	65	11	20	230	55	18	9	244	39	9	17	261
Huang (45)	2014	Taiwan	324	Middlebrook 7H10		clinical isolates	case control	217	0	27	39	248	0	3	39	182	0	28	39
Kumar (46)	2014	India	141	MGIT 960		clinical isolates	cross sectional	65	0	5	71	62	0	2	77	54	0	4	83
Luetkemeyer (47)	2014	South Africa	282	MGIT SIRE	+ &−	clinical specimens	cross sectional	24	3	4	257	22	12	0	248	ND	ND	ND	ND
Raizada (48)	2014	India	248	L-J PM	+	clinical specimens	cross sectional	133	2	52	61	127	7	9	105	ND	ND	ND	ND
Gupta (49)	2015	India	89	MGIT 960		clinical isolates	cross sectional	13	2	1	73	4	1	1	83	5	0	0	84

Abbreviations: TR = true resistance; FR = false resistance; FS = false susceptibility; TS = true susceptibility; INH = Isoniazid; RIF = Rifampicin; MDR = Multi drug resistance; BACT 460 = Radiometric BACTEC 460;L-J PM = Proportion method on Lowenstein-Jensen medium, MGIT = Mycobacterium growth indicator tube; SIRE = streptomycin, INH, RMP, and ethambutol; ND = No data in study report.

### Quality Assessment

**Fig 2:**
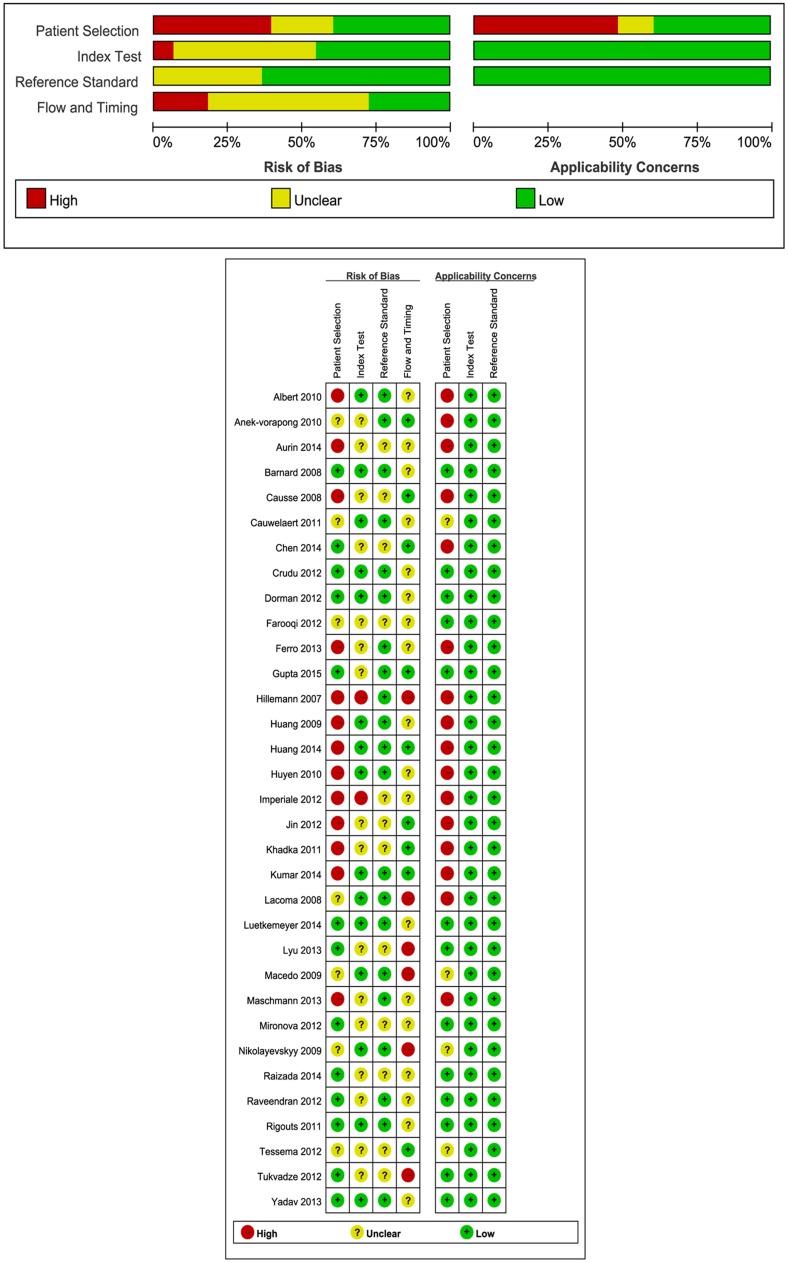
Quality assessment of included studies. Quality Assessment of Diagnostic Accuracy Studies version 2: risk of bias and applicability concerns summary of MTBDR*plus* for the detection of drug resistance.

A quality assessment of all of the included studies is illustrated in Fig 2. Most of the included studies were at either high risk or unclear risk bias in “patient selection” and “flow and timing” domains of QUARDAS-2 due to lack of detail regarding timing, inconsecutive, or nonrandom patient selection and blinding. A total of 13 (32.5%) studies were at low risk, 7 studies (17.5%) were of unclear risk, and 20 studies (50%) were at high risk for patient selection bias. A total of 24 studies (60%) were at high risk for flow and timing bias, resulting from the fact that not all selected patients were included in the diagnostic analysis and the patients did not receive the same gold standard DST. Most of the studies were at either low or unclear risk for index test and reference standard bias. Regarding applicability, half of the studies were at high risk for patient selection; however, all selected studies (n = 40, 100%) were at low risk of index test and the reference standard. In summary, patient selection was the most high-risk bias and high-risk applicability concerns.

### Diagnostic Accuracy

#### Detection of INH resistance

**Fig 3:**
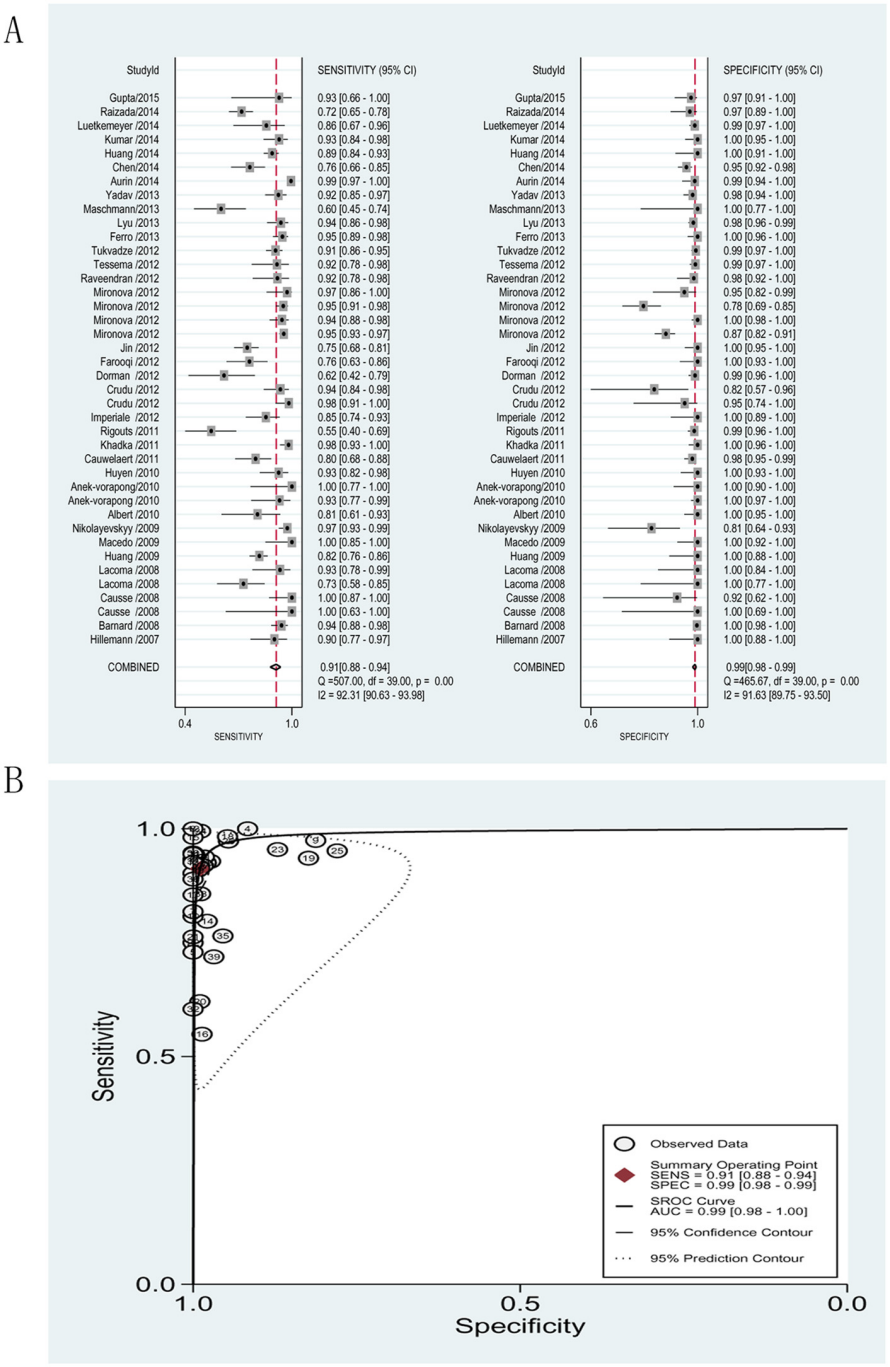
Forest plots of the pooled sensitivity and specificity and SROC curve of MTBDR*plus* for detection of isoniazid drug susceptibility. (A). Forest plots of the pooled sensitivity and specificity. Each solid square represents an individual study. Error bars represent 95% CI. Diamond indicates the pooled sensitivity and specificity for all of the studies. (B). SROC curve.

The pooled sensitivity and specificity for detection of resistance to INH were 0.91 (95% CI = 0.88–0.94) and 0.99 (95% CI = 0.98–0.99), respectively. The PLR and NLR were 85.03 (95% CI = 44.16–163.74) and 0.09 (95% CI = 0.08–0.12), respectively. The DOR was 958.40 (95% CI = 469.52–1956.34) and the AUC was 0.99 (95% CI = 0.98–1.00), indicating a high level of overall accuracy (Fig 3, see also Table 2).

**Table 2:** Summarized diagnostic accuracy of GenoType MTBDR*plus*.

Drug	Se (95% CI)	Sp (95% CI)	PLR (95% CI)	NLR (95% CI)	DOR (95% CI)
INH	0.91 (0.88–0.94)	0.99 (0.98–0.99)	85.03 (44.16–163.74)	0.09(0.08–0.12)	958.40 (469.52–1956.34)
RIF	0.96(0.95–0.97)	0.98(0.97–0.99)	59.44(35.51–99.51)	0.04(0.03–0.05)	1635.08(838.31–3196.78)
MDR	0.91(0.86–0.94)	0.99(0.99–1.00)	173.38(73.90–406.8)	0.09(0.06–0.15)	1838.91(653.30–5176.16)

Abbreviations: INH = isoniazid; RIF = rifampicin; MDR = multi drug resistance; Se = sensitivity; Sp = specificity; PLR = positive likelihood ratio; NLR = negative likelihood ratio; DOR = diagnostic odds ratio; CI = confidence interval.

#### Detection of RIF resistance

**Fig 4:**
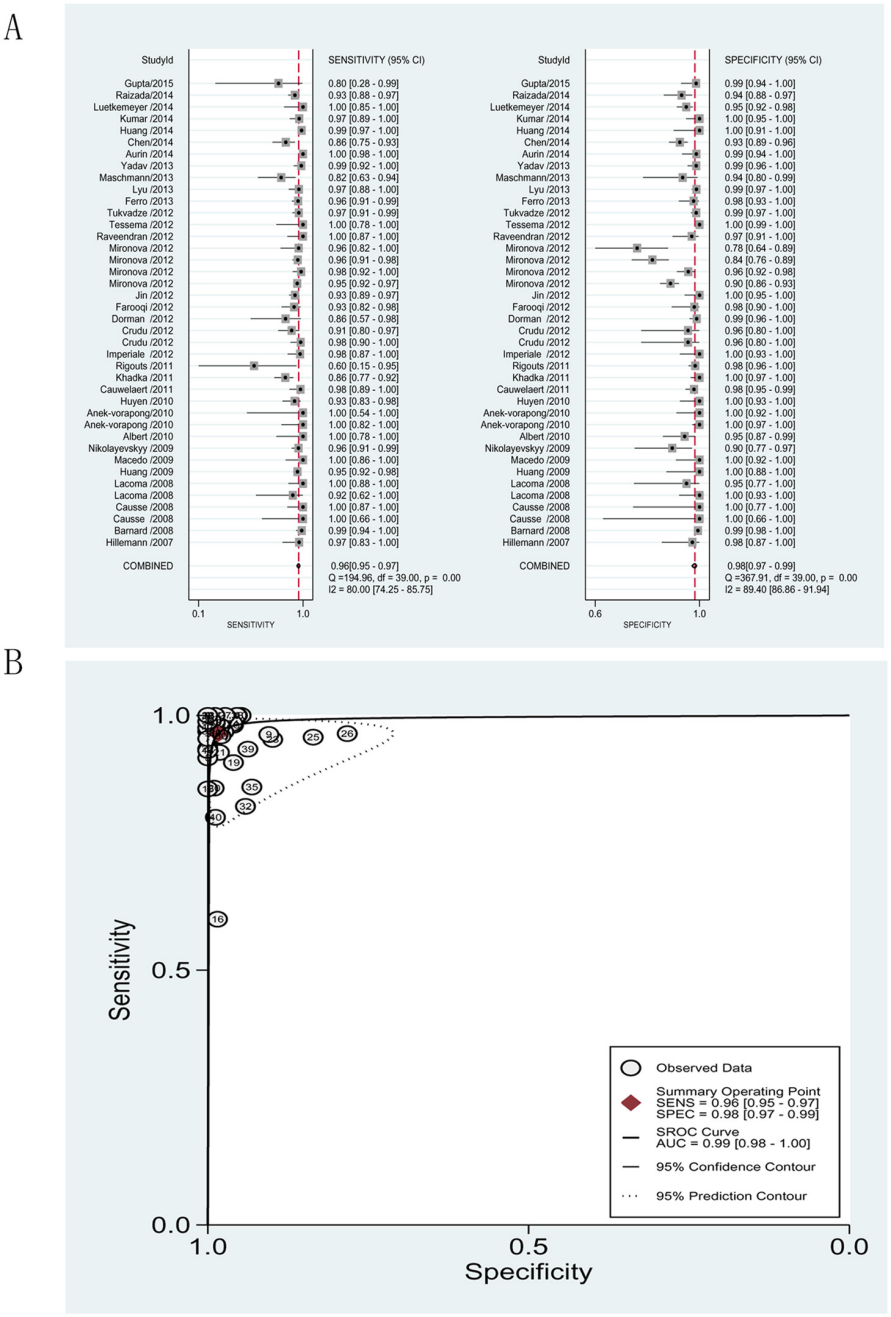
Forest plots of the pooled sensitivity and specificity and SROC curve of MTBDR*plus* for detection of rifampicin drug susceptibility. (A). Forest plots of the pooled sensitivity and specificity. Each solid square represents an individual study. Error bars represent 95% CI. Diamond indicates the pooled sensitivity and specificity for all of the studies. (B). SROC curve.

The pooled sensitivity and specificity for detection of resistance to RIF were 0.96 (95% CI = 0.95–0.97) and 0.98 (95% CI = 0.97–0.99), respectively. The PLR and NLR were 59.44 (95% CI = 35.51–99.51) and 0.04 (95% CI = 0.03–0.05), respectively. The DOR was 1635.08 (95% CI = 838.31–3196.78) and the AUC was 0.99 (95% CI = 0.98–1.00), indicating a high level of overall accuracy (Fig 4, see also Table 2).

#### Detection of MDR

**Fig 5:**
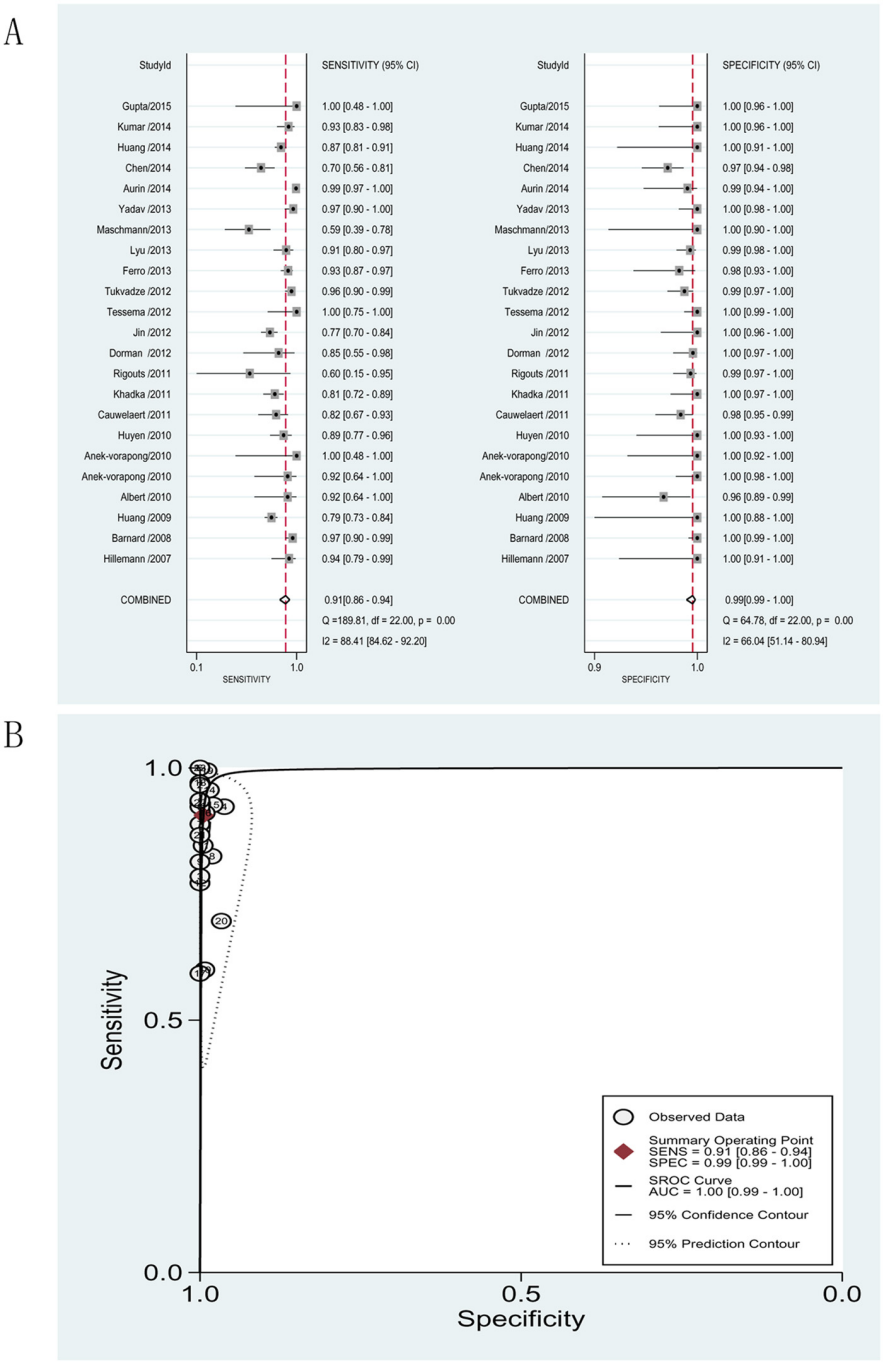
Forest plots of the pooled sensitivity and specificity and SROC curve of MTBDR*plus* for detection of multidrug-resistant tuberculosis. (A). Forest plots of the pooled sensitivity and specificity. Each solid square represents an individual study. Error bars represent 95% CI. Diamond indicates the pooled sensitivity and specificity for all of the studies. (B). SROC curve.

The pooled sensitivity and specificity for detection of MDR were 0.91 (95% CI = 0.86–0.94) and 0.99 (95% CI = 0.99–1.00), respectively. The PLR and NLR were 173.38 (95% CI = 73.90–406.8) and 0.09 (95% CI = 0.06–0.15), respectively. The DOR was 1838.91(95% CI = 653.30–5176.16) and the AUC was 1.00 (95% CI = 0.99–1.00), indicating a good level of overall accuracy (Fig 5, see also Table 2).

### Heterogeneity

Significant heterogeneity was observed when we pooled sensitivity, specificity, PLR, NLR, and DOR of selected studies. The heterogeneity test results of sensitivity and specificity are illustrated in the forest plots (Figs 3, 4 and 5). The Spearman correlation coefficient between the logit of sensitivity and logit of 1-specificity was used to assess the threshold/cut-off effect. The Spearman correlation coefficient (*p* value) in detecting resistance to INH, RIF and MDR was 0.153 (*p* = 0.345), 0.017 (*p* = 0.915), -0.227 (*p* = 0.298), respectively. This indicated that the heterogeneity might not be due to threshold/cut-off effect. To assess for causes of variations other than threshold, we performed subgroup analysis with the GenoType MTBDR*plus* assay performed directly on clinical samples or indirectly on clinical isolates, in either solid or liquid medium.

### Subgroup Analyses

According to the type of specimen as well as medium, 40 studies were included in the subgroup analyses. Pooled sensitivity, specificity, PLR, NLR, and DOR for INH, RIF, and MDR are presented in Tables 3 and 4. We found significant heterogeneity for most of these measures, except for only clinical isolates were pooled when using GenoType MTBDR*plus* to detect specificity of MDR (I^2^ = 45.5%, *p* = 0.06).

**Table 3:** Subgroup analyses by specimen type.

Drug	Specimen type	Se (95% CI)	Sp (95% CI)	PLR (95% CI)	NLR (95% CI)	DOR (95% CI)
INH	Clinical specimens	0.90(0.85–0.94)	0.98(0.96–0.99)	52.73(25.18–110.44)	0.10(0.06–0.16)	534.62(233.67–1223.16)
	Clinical isolates	0.93(0.88–0.96)	1.00(0.98–1.00)	282.13(44.81–1776.28)	0.07(0.04–0.12)	4045.75(681.59–24014.72)
RIF	Clinical specimens	0.97(0.94–0.98)	0.97(0.96–0.98)	37.31(22.36–62.24)	0.03(0.02–0.06)	1105.23(469.70–2600.63)
	Clinical isolates	0.96(0.93–0.98)	1.00(0.97–1.00)	411.81(35.54–4771.87)	0.04(0.02–0.07)	10169.89(909.58–1.1e+05)
MDR	Clinical specimens	0.92(0.83–0.96)	0.99(0.98–1.00)	114.91(49.58–266.34)	0.08(0.04–0.17)	1382.17(367.13–5203.57)
	Clinical isolates	0.86(0.81–0.90)	1.00(0.78–1.00)	7023.24(3.04–1.6e+07)	0.14(0.10–0.19)	51193.70(22.94–1.1e+08)

Abbreviations: INH = isoniazid;RIF = rifampicin; MDR = multi drug resistance; Se = sensitivity; Sp = specificity; PLR = positive likelihood ratio; NLR = negative likelihood ratio; DOR = diagnostic odds ratio; CI = confidence interval.

**Table 4:** Subgroup analyses by medium type.

Drug	Medium type	Se (95% CI)	Sp (95% CI)	PLR (95% CI)	NLR (95% CI)	DOR (95% CI)
INH	Solid medium	0.90(0.83–0.95)	0.98(0.96–0.99)	54.99(22.62–133.65)	0.10(0.06–0.18)	549.11(191.34–1575.84)
	Liquid medium	0.92(0.88–0.95)	0.99(0.98–1.00)	122.57(49.31–304.68)	0.08(0.05–0.12)	1502.66(571.95–3947.84)
RIF	Solid medium	0.95(0.92–0.97)	0.98(0.95–0.99)	40.19(19.61–82.37)	0.05(0.03–0.08)	796.57(312.67–2029.40)
	Liquid medium	0.98(096–0.99)	0.99(0.97–1.00)	85.44(36.88–197.94)	0.03(0.01–0.04)	3387.38(1122.12–10225.61)
MDR	Solid medium	0.87(0.77–0.93)	0.99(0.98–1.00)	105.81(40.51–276.36)	0.13(0.07–0.24)	816.79(229.92–2901.68)
	Liquid medium	0.94(0.90–0.97)	0.997(0.992–0.999)	167.98(75.45–373.85)	0.08(0.05–0.12)	2111.60(771.45–5780.0)

Abbreviations: INH = isoniazid;RIF = rifampicin; MDR = multi drug resistance;Se = sensitivity; Sp = specificity; PLR = positive likelihood ratio; NLR = negative likelihood ratio; DOR = diagnostic odds ratio; CI = confidence interval.

## Discussion

Molecular drug susceptibility testing for *M*. *tuberculosis* has garnered strong research interest worldwide. To that end, we focused on the GenoType MTBDR*plus* assay which has been recommended by the WHO to rapidly screen patients at risk of MDR-TB [10]. MTBDR*plus* assay is now used routinely in many countries due to its shorter turnaround time, thus a more effective procedure. The direct use of the assay on clinical specimens is another key advantage, as this precludes waiting for cultures to grow. Different from other rapid molecular tests such as INNO-LiPA and GeneXpert, MTBDR*plus* assay not only detects RIF resistance, but also INH resistance. Although RIF resistance may be regarded as a surrogate for MDR to some extent, there are still some RIF-monoresistant TB strains that are not MDR. Thus, the inclusion of testing mutations that cause INH resistance is highly desirable, especially in settings with relatively low MDR-TB prevalence [50]. Furthermore, the MTBDR*plus* assay has been the most cost-effective rapid test for Asian populations in current practice [13], and its implementation to detect MDR-TB can improve clinical outcomes significantly in some settings [51]. Recently, studies focusing on the diagnostic accuracy of GenoType MTBDR*plus* were conducted in many settings, but with inconsistent results. The aim of this meta-analysis was to evaluate the diagnostic accuracy of GenoType MTBDR*plus* for direct detection of resistance to RIF and INH compared with conventional reference methods.

In the literature there are three meta-analyses in which the GenoType MTBDR*plus* assay has been assessed. The first analysis, performed in 2008, evaluated the performance of both the old GenoType MTBDR and GenoType MTBDR*plus*, with analysis of only five MTBDR*plus* studies for the determination of INH and RIF resistance [12]. The second analysis, published in 2009, evaluated the performance of four direct-testing methods, including GenoType MTBDR*plus*, also with analysis of only five studies for the determination of MDR [50]. The recently reported systematic review, published in 2015, focused on four main molecular diagnostic tests for antibiotic resistance in *M*. *tuberculosis*, including GenoType MTBDR*plus*, and only evaluated the assay on clinical specimens and could not perform subgroup analysis to investigate the potential causes of heterogeneity due to the small number included studies [13]. To the best of our knowledge, the present meta-analysis, with 40 studies included, is the first study that has comprehensively evaluated the overall diagnostic accuracy of the GenoType MTBDR*plus* assay in detecting drug resistance of RIF, INH, and MDR.

In our meta-analysis, GenoType MTBDR*plus* showed excellent pooled sensitivity and specificity for detection of resistance to INH (91%, 99%), RIF (96%, 98%), and MDR (91%, 99%), with lower and more inconsistent sensitivity than specificity. While specificity did not vary across subgroups, sensitivity was slightly higher when only DST of studies based on liquid medium was pooled (INH 92%, RIF 98%, MDR 94%). When compared with the previously published meta-analyses, the pooled sensitivity was also found to be more variable and lower than specificity, which varied from 84% to 96% for INH and 96% to 99% for RIF [12, 13, 52]. This may be partially attributed to the limitations of molecular methods for the detection of first line drug resistance, that 5% of RIF-resistant *M*. *tuberculosis* strains and 10–25% of low-level INH-resistant strains have no known resistance mutations [53, 54].

The DOR is defined as the ratio of the odds of the test being positive for a patient with or without disease [55], and is an indicator of diagnostic accuracy that combines the data from sensitivity and specificity into a single variate. The value of a DOR ranges from 0 to infinity, with higher values indicating higher accuracy. This meta-analysis showed that GenoType MTBDR*plus* had very high mean DOR and large AUC values, indicating a high value of overall accuracy for the detection of MDR. Because of the limitations of SROC and DOR in clinical practice, the likelihood ratios (LRs) are of more clinical significance [56]. A very high PLR and a very low NLR for the detection resistance of INH, RIF, and MDR in our study indicated an excellent ability to both confirm and exclude the presence of drug resistance. Although in the present analysis, indices such as AUC, DOR, PLR, and NLR showed good diagnostic accuracy of GenoType MTBDR*plus* assay, the confidence intervals for the PLR and the DOR were wide for all included studies due to high sample variation and there was significant heterogeneity in the measures.

The purpose of a meta-analysis is not only to compute a single summary measure, but also to explore the reasons for heterogeneity [57]. We found significant heterogeneity for sensitivity, specificity, PLR, NLR, and DOR among the studies analyzed, except for only clinical isolates were pooled when using GenoType MTBDR*plus* to detect specificity of MDR (I^2^ = 45.5%, *p* = 0.06). The Spearman correlation coefficient between the logit of sensitivity and logit of 1-specificity was not significant, indicating that the heterogeneity was not caused by threshold/cut-off effect. Thus, subgroup analyses were performed to test for causes of variations other than threshold effect. The results suggested that the sample type could partly explain the heterogeneity. Even so, the considerable heterogeneity in the results remained unexplained, which may be caused by variations in the study, patient selection, sample collection method (consecutive or random collection of samples), and/or geographic and genetic variations in the distribution of drug-resistant strains of *M*. *tuberculosis* [58, 59].

Our meta-analysis had several strengths. First, we performed a standard protocol to carry out the meta-analysis, including a comprehensive search strategy [60]. Second, two reviewers independently carried out various stages of the process, including article selection, data extraction, and quality assessment, and disagreements were resolved by consensus. Third, we used rigorous statistical methods for data analysis, including SROC analyses, quality assessment relying on QUADAS-2, as methods for exploring heterogeneity. Moreover, the present meta-analysis updates previous estimates on the performance of the MTBDR*plus* test for identifying resistance of first-line anti-TB drugs. Compared with the recently published comprehensive systematic review [13], our study showed similar pooled specificity, but higher pooled sensitivity for detecting both RIF and INH resistance (97% *versus* 94.6%; and 90% *versus* 83.4%, respectively) directly on clinical specimens. The DOR, as an indicator of diagnostic accuracy, was also much higher in the current study than previously shown for detecting RIF resistance (1105.23 *versus* 666). The better diagnostic accuracy found in our study may provide more powerful evidence for routine clinical application of GenoType MTBDR*plus* assay.

However, our meta-analysis also had several limitations. First, sampling methods, blinding strategies and population (e.g. severity of disease or treatment status) were unclear in most of the included studies. Inappropriate sampling methods can generate selection bias which may result in high levels of sample variation and wide confidence intervals. The lack of blinding when interpreting index and reference test results may result in overestimating accuracy [61]. Second, an obvious limitation was the lack of data on cost-effectiveness, feasibility, patient management and treatment outcomes, and how much value they contributed to existing diagnostic and treatment regimens beyond conventional DST methods. Third, the present authors only included studies published in English, and some studies missing data in 2 by 2 tables were excluded since the authors could not be contacted. As currently available statistical approaches for publication bias are not recommended for diagnostic meta-analysis, we did not use funnel plots and regression tests to assess publication bias [62], and it is therefore difficult to rule out potential publication bias in our meta-analysis.

Furthermore, there were not enough studies in the literature for us to acquire adequate data to stratify by smear status, as smear-negative patients would be most likely to benefit from using molecular methods. Until now, it seems there is still a great challenge to rapidly and reliably identify *M*. *tuberculosis* in smear-negative samples, especially in human immunodeficiency virus (HIV)-infected patients. *M*. *tuberculosis* is the most prevalent opportunistic infection and cause of the death for HIV-infected patients, whose smear-positivity of *M*. *tuberculosis* can be as low as 20% [63]. To overcome this limitation, the revised version 2.0 of MTBDR*plus* was released in 2011 with reported improved diagnostic accuracy in detecting *M*. *tuberculosis* and their resistance status against RIF and INH in AFB-negative specimens [31, 64], further supporting the ability to use this assay in smear-negative samples.

In general, although GenoType MTBDR*plus* test showed good accuracy for INH, RIF, and MDR drug resistance detection in this meta-analysis, some important issues remain to be addressed. In recent years, several studies showed that RIF resistance can be regarded as a proxy for MDR in different settings [65, 66]. Arentz *et al*. performed a systematic review to evaluate six different WHO-endorsed rapid tests for RIF resistance detection [67], and determined that these tests for RIF resistance can accurately predict MDR-TB in areas with high prevalence, but not in areas with low prevalence of RIF resistance. Compared with other tests, GenoType MTBDR*plus* had the lowest PPV at prevalence rates of 15% and 3% for RIF resistance which meant the higher false positive rates for detecting RIF resistance and MDR-TB. However, these results relied on an assumption that RIF resistance was strongly correlated with MDR. In fact, this correlation may vary in different settings [50]. Future studies should focus on the diagnostic accuracy of rapid tests in areas with different prevalence rates of RIF resistance in order to determine the threshold that constitutes RIF resistance is as a sufficient marker for MDR-TB.

In addition to rapid detection of MDR-TB, there is also an urgent need for rapid and accurate tests for extensively drug-resistant tuberculosis (XDR-TB). As a serious threat to public health, XDR-TB is caused by strains of *M*. *tuberculosis* that are resistant to INH, RIF, and any of the fluoroquinolones (FLQs) and at least one second-line injectable agent (SLIDs; i.e. amikacin, kanamycin or capreomycin) [68]. XDR-TB has now been detected in more than 90 countries and nearly 10% of MDR-TB cases are also XDR-TB cases [2]. A recently published systematic review found GenoType MTBDR*sl*, the only commercially-available molecular routine test to detect second-line anti-TB drug resistance, had good accuracy for detecting drug resistance to FLQs, amikacin and capreomycin, but may not be an appropriate choice for kanamycin and ethambutol due to poor sensitivity [69]. Future studies that test the accuracy of the MTBDR*sl* in different laboratory settings are necessary. Furthermore, differences should be accounted for geographical regions, special patient populations (for example, pediatric or HIV/TB co-infected patients), and should also assess the effect of MTBDR*sl* implementation on cost-effectiveness and clinical outcomes. Future molecular tests for XDR-TB should have additional genetic targets beyond *gyrA*, *rrs* and *embB*. Rapid and accurate detection of MDR-TB and XDR-TB is important in improving patient care and decreasing transmission.

In conclusion, the present meta-analysis showed that GenoType MTBDR*plus* assay had good accuracy for detecting drug resistance to INH, RIF, and MDR of *M*. *tuberculosis*, suggesting that it has good utility as a rapid screening molecular tool. Further studies are needed to compare the accuracy of the MTBDR*plus* assay in smear-positive *versus* smear-negative specimens and pulmonary *versus* extra-pulmonary cases, and to evaluate the utility of this assay in HIV/TB co-infection. MTBDR*plus* assay might be a good alternative to conventional drug susceptibility tests in clinical practice.

## Supporting Information

S1 File PRISMA Checklist. (DOC)

S1 Table The 20 full-text excluded studies with the reasons for exclusion. (DOC)
