# Supplementary material for: GenoType MTBDRplus Assay for Rapid Detection of Multidrug Resistance in Mycobacterium tuberculosis: A Meta-Analysis
Source: PLoS One. 2016 Mar 2;11(3):e0150321. doi: 10.1371/journal.pone.0150321 (PMC4774872; doi:10.1371/journal.pone.0150321)
Supplement: S1 Table — (DOC) [file pone.0150321.s002.doc]

| **S1 Table. The 20 full-text excluded studies with the reasons for exclusion.** | | | |
| --- | --- | --- | --- |
| Reasons for exclusion | First author | Publication year | PMID |
| Comparision studies  (n=4) | Al-Mutairi NM | 2011 | 21276306 |
|  | Banu S | 2014 | 24172155 |
|  | Kurup R | 2013 | 24564061 |
|  | Rufai SB | 2014 | 24648554 |
| Sequencing or 1.0 as standards  (n=5) | Dinic L | 2012 | 22740709 |
|  | Barnard M | 2012 | 22972826 |
|  | Hauck Y | 2009 | 19520715 |
|  | Minime-Lingoupou F | 2010 | 20487620 |
|  | Kambli P | 2015 | 25749461 |
| Not compared with reference testing(n=5) | Brossier F | 2009 | 19146757 |
|  | Singhal R | 2015 | 25657156 |
|  | Sharma S | 2014 | 25118735 |
|  | Zhang L | 2011 | 20852939 |
|  | Singhal R | 2014 | 25488443 |
| Could not extract 2×2 tables  （n=6） | Akpaka PE | 2008 | 18701663 |
|  | Aubry A | 2014 | 24743770 |
|  | Duo L | 2011 | 22021920 |
|  | Folkvardsen DB | 2013 | 23447641 |
|  | Macedo R | 2009 | 19401789 |
|  | Scott LE | 2011 | 21814495 |
